# First Observation of Hemoglobin Jabalpur [Beta 3 (NA3) Leu>Pro] in the Turkish Population

**DOI:** 10.4274/tjh.2014.0027

**Published:** 2014-09-05

**Authors:** Ayfer Çolak, Burak Toprak, Kanay Yararbaş, Fatma Akyol, Cengiz Ceylan

**Affiliations:** 1 Hemoglobinopathy, Hb Jabalpur, High-performance liquid chromatography; 2 Düzen Laboratory Group, İstanbul, Turkey; 3 Tepecik Teaching and Research Hospital, Clinic of Hematology, İzmir, Turkey

**Keywords:** Hemoglobinopathy, Hb Jabalpur, High-performance liquid chromatography

## TO THE EDITOR

Hemoglobin Jabalpur [beta 3(NA3) Leu>Pro] is a rare hemoglobin variant previously described in the HBVar database of the Globin Gene Server [1]. In the present paper we report Hb Jabalpur identified in a Turkish family. This is the first report of Hb Jabalpur in the Turkish population.

The patient was an asymptomatic 19-year-old male who presented to the outpatient hematology clinic for further evaluation of mild anemia. On physical examination the patient’s liver and spleen were not palpable. The patient’s complete blood cell count showed a hemoglobin level of 116 g/L (Table 1). Cation-exchange high-performance liquid chromatography, which was performed using the Primus Ultra2 Hb variant analyzer (Trinity Biotech, Dublin, Ireland), showed abnormal hemoglobin amounting to 30.9% of total hemoglobin. Hb Jabalpur was eluted immediately before Hb A0 in the Hb A window.

**Figure 1:**
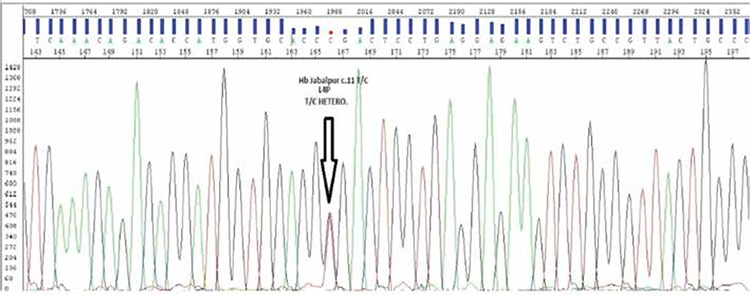
DNA sequencing data showing Hb Jabalpur.

Using direct Sanger sequencing (ABI 3130 Genetic Analyzer, Applied Biosystems, Foster City, CA, USA), we characterized the Hb variant as resulting from a CTG>CCG (c.11T>C, p.L4P, rs63750720, NCBI refSeq: NG_000007.3) replacement at codon 3 of the β-globin chain, corresponding to a Leu→Pro amino acid substitution (Figure 1). This mutation is not listed in the Human Gene Mutation Database as a disease-causing mutation, while it is present in the Hemoglobin Variation Database (HbVar: A Database of Human Hemoglobin Variants and Thalassemias) as Hb variant Jabalpur. Informed consent was obtained.

 The parents of the patient were also available for evaluation. DNA sequencing and chromatography analysis revealed that the mother also had Hb Jabalpur. The mother was found to have mild anemia (Hb: 107g/L) and no clinical symptoms were detected (Table 1).

The index patient and his mother were living in İzmir, in the Aegean region of Turkey. Hb Jabalpur was detected in another patient of Pakistani origin with compound heterozygous beta zero thalassemia, and moderate anemia (Hb: 97g/L) was also observed in that patient (Gallivan M 2010, personal communication). In the present report, both the index patient and his mother presented with mild anemia. It is known that the prevalence of the beta-thalassemia carrier state is high in Turkey; furthermore, several rare hemoglobin variants were previously reported. Hb Jabalpur and many rare hemoglobin variants generally do not produce clinical symptoms, but the consequences of the interaction between Hb variants and beta-thalassemia may be clinically important.

## CONFLICT OF INTEREST STATEMENT

The authors of this paper have no conflicts of interest, including specific financial interests, relationships, and/ or affiliations relevant to the subject matter or materials included.

## Figures and Tables

**Table 1:**
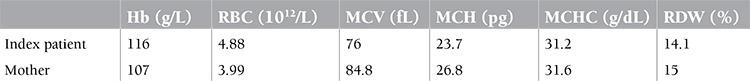
Hematological data of index patient and his mother.
